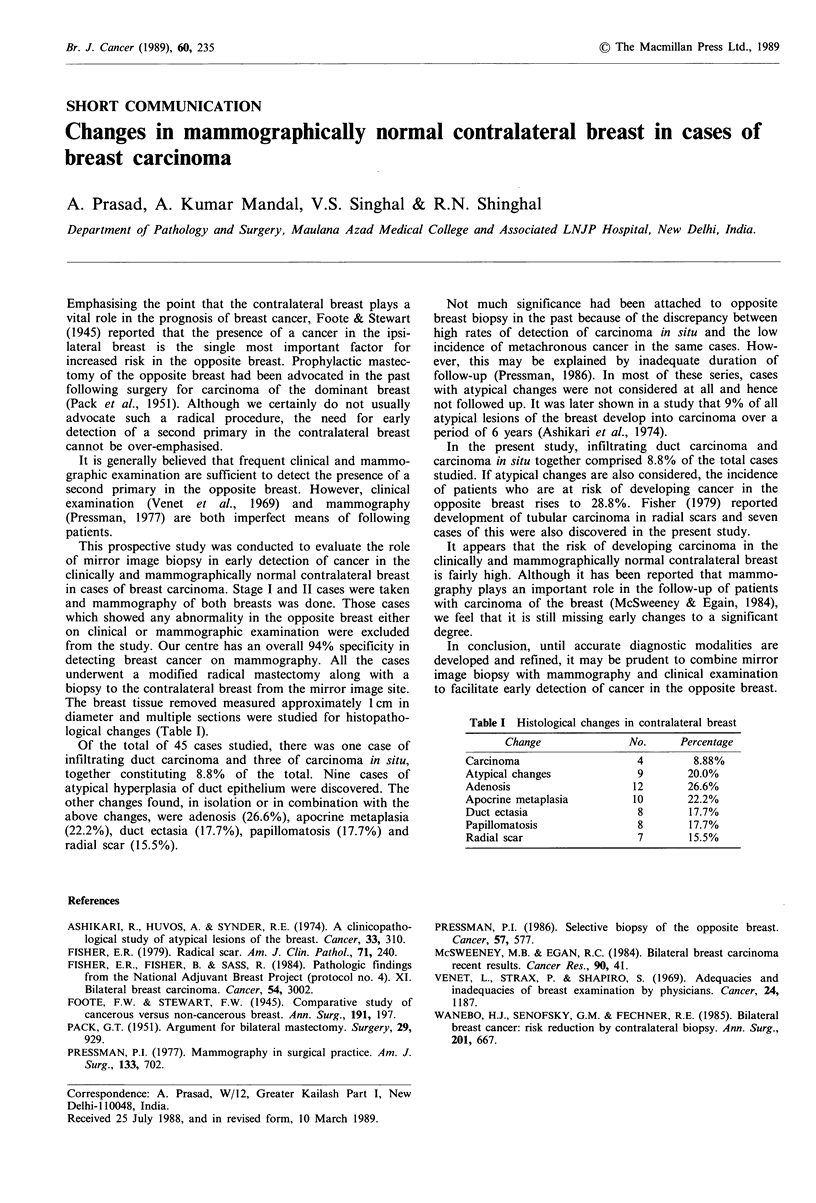# Changes in mammographically normal contralateral breast in cases of breast carcinoma.

**DOI:** 10.1038/bjc.1989.259

**Published:** 1989-08

**Authors:** A. Prasad, A. K. Mandal, V. S. Singhal, R. N. Shinghal

**Affiliations:** Department of Pathology and Surgery, Maulana Azad Medical College, New Delhi, India.


Br. J. Cancer (1989), 60, 235 © The Macmillan Press Ltd., 1989

SHORT COMMUNICATION

### Changes in mammographically normal contralateral breast in cases of breast carcinoma

A. Prasad, A. Kumar Mandal, V.S. Singhal & R.N. Shinghal

*Department of Pathology and Surgery, Maulana Azad Medical College and Associated LNJP Hospital, New Delhi, India.*

Emphasising the point that the contralateral breast plays a vital role in the prognosis of breast cancer, Foote & Stewart (1945) reported that the presence of a cancer in the ipsilateral breast is the single most important factor for increased risk in the opposite breast. Prophylactic mastectomy of the opposite breast had been advocated in the past following surgery for carcinoma of the dominant breast (Pack *et al.*, 1951). Although we certainly do not usually advocate such a radical procedure, the need for early detection of a second primary in the contralateral breast cannot be over-emphasised.

It is generally believed that frequent clinical and mammographic examination are sufficient to detect the presence of a second primary in the opposite breast. However, clinical examination (Venet *et al.*, 1969) and mammography (Pressman, 1977) are both imperfect means of following patients.

This prospective study was conducted to evaluate the role of mirror image biopsy in early detection of cancer in the clinically and mammographically normal contralateral breast in cases of breast carcinoma. Stage I and II cases were taken and mammography of both breasts was done. Those cases which showed any abnormality in the opposite breast either on clinical or mammographic examination were excluded from the study. Our centre has an overall 94% specificity in detecting breast cancer on mammography. All the cases underwent a modified radical mastectomy along with a biopsy to the contralateral breast from the mirror image site. The breast tissue removed measured approximately 1 cm in diameter and multiple sections were studied for histopathological changes (Table I).

Of the total of 45 cases studied, there was one case of infiltrating duct carcinoma and three of carcinoma *in situ*, together constituting 8.8% of the total. Nine cases of atypical hyperplasia of duct epithelium were discovered. The other changes found, in isolation or in combination with the above changes, were adenosis (26.6%), apocrine metaplasia (22.2%), duct ectasia (17.7%), papillomatosis (17.7%) and radial scar (15.5%).

Not much significance had been attached to opposite breast biopsy in the past because of the discrepancy between high rates of detection of carcinoma *in situ* and the low incidence of metachronous cancer in the same cases. However, this may be explained by inadequate duration of follow-up (Pressman, 1986). In most of these series, cases with atypical changes were not considered at all and hence not followed up. It was later shown in a study that 9% of all atypical lesions of the breast develop into carcinoma over a period of 6 years (Ashikari *et al.*, 1974).

In the present study, infiltrating duct carcinoma and carcinoma *in situ* together comprised 8.8% of the total cases studied. If atypical changes are also considered, the incidence of patients who are at risk of developing cancer in the opposite breast rises to 28.8%. Fisher (1979) reported development of tubular carcinoma in radial scars and seven cases of this were also discovered in the present study.

It appears that the risk of developing carcinoma in the clinically and mammographically normal contralateral breast is fairly high. Although it has been reported that mammography plays an important role in the follow-up of patients with carcinoma of the breast (McSweeney & Egain, 1984), we feel that it is still missing early changes to a significant degree.

In conclusion, until accurate diagnostic modalities are developed and refined, it may be prudent to combine mirror image biopsy with mammography and clinical examination to facilitate early detection of cancer in the opposite breast.

**Table I** Histological changes in contralateral breast

| *Change* | *No.* | *Percentage* |
|---|---|---|
| Carcinoma | 4 | 8.88% |
| Atypical changes | 9 | 20.0% |
| Adenosis | 12 | 26.6% |
| Apocrine metaplasia | 10 | 22.2% |
| Duct ectasia | 8 | 17.7% |
| Papillomatosis | 8 | 17.7% |
| Radial scar | 7 | 15.5% |

### References

ASHIKARI, R., HUVOS, A. & SYNDER, R.E. (1974). A clinicopathological study of atypical lesions of the breast. *Cancer*, **33**, 310.

FISHER, E.R. (1979). Radical scar. *Am. J. Clin. Pathol.*, **71**, 240.

FISHER, E.R., FISHER, B. & SASS, R. (1984). Pathologic findings from the National Adjuvant Breast Project (protocol no. 4). XI. Bilateral breast carcinoma. *Cancer*, **54**, 3002.

FOOTE, F.W. & STEWART, F.W. (1945). Comparative study of cancerous versus non-cancerous breast. *Ann. Surg.*, **191**, 197.

PACK, G.T. (1951). Argument for bilateral mastectomy. *Surgery*, **29**, 929.

PRESSMAN, P.I. (1977). Mammography in surgical practice. *Am. J. Surg.*, **133**, 702.

PRESSMAN, P.I. (1986). Selective biopsy of the opposite breast. *Cancer*, **57**, 577.

McSWEENEY, M.B. & EGAN, R.C. (1984). Bilateral breast carcinoma recent results. *Cancer Res.*, **90**, 41.

VENET, L., STRAX, P. & SHAPIRO, S. (1969). Adequacies and inadequacies of breast examination by physicians. *Cancer*, **24**, 1187.

WANEBO, H.J., SENOFSKY, G.M. & FECHNER, R.E. (1985). Bilateral breast cancer: risk reduction by contralateral biopsy. *Ann. Surg.*, **201**, 667.

Correspondence: A. Prasad, W/12, Greater Kailash Part I, New Delhi-110048, India.

Received 25 July 1988, and in revised form, 10 March 1989.